# Highly Sensitive Detection of NO_2_ by Au and TiO_2_ Nanoparticles Decorated SWCNTs Sensors

**DOI:** 10.3390/s20010012

**Published:** 2019-12-18

**Authors:** Ada Fort, Enza Panzardi, Ammar Al-Hamry, Valerio Vignoli, Marco Mugnaini, Tommaso Addabbo, Olfa Kanoun

**Affiliations:** 1 Department of Information Engineering and Mathematical Sciences, University of Siena, 53100 Siena, Italy; vignoli@diism.unisi.it (V.V.); addabbo@diism.unisi.it (T.A.); 2 Chair Measurement and Sensor Technology, Department of electrical Engineering and Information Technology, Chemnitz University of Technology, 09107 Chemnitz, GermanyOlfa.Kanoun@etit.tu-chemnitz.de (O.K.)

**Keywords:** CNTs gas sensors, nanomaterials, chemoresistors

## Abstract

The aim of this work is to investigate the gas sensing performance of single wall carbon nanotubes (SWCNTs)-based conductive sensors operating at low–medium temperatures (<250 °C). The investigated sensing films consists of an SWCNT network obtained by drop-casting a SWCNT suspension. Starting from this base preparation, different sensing devices were obtained by decorating the SWCNT network with materials suitable for enhancing the sensitivity toward the target gas. In particular, in this paper, nano-particles of gold and of TiO_2_ were used. In the paper, the performance of the different sensing devices, in terms of response time, sensitivity toward NO_2_ and cross-sensitivity to O_2_, CO and water vapor, were assessed and discussed. Sensors based on decorated SWCNT films showed high performance; in particular, the decoration with Au nano-particles allows for a large enhancement of sensitivity (reaching 10%/1 ppm at 240 °C) and a large reduction of response time. On the other hand, the addition of TiO_2_ nanoparticles leads to a satisfactory improvement of the sensitivity as well as a significant reduction of the response time at moderate temperatures (down to 200 °C). Finally, the suitability of using Au decorated SWCNTs-based sensors for room temperature sensing is demonstrated.

## 1. Introduction

Carbon nanotubes (CNTs), since their discovery in 1991 by Iijima [1], have deeply attracted the interest of researchers, enough to play a key role in nanotechnology nowadays [2,3,4,5], and have found employments in a wide range of engineering applications. In particular, CNTs are part of the fullerene family, which is one of the allotrope forms of the carbon element [6]. It is possible to distinguish two typologies of nanotubes: single-walled carbon nanotubes (SWCNTs) and multi-walled carbon nanotubes (MWCNTs) [7]. SWCNTs consist of a single atom-thick layer of graphene wound on itself in a cylindrical shape, whereas MWCNTs can be seen as multiple concentric SWCNTs structures. Their electronic behavior depends heavily on their size and chirality, CNTs in fact can behave as semiconductors (with a band gap that can be easily modified as it depends, for instance, on the tube diameter) or, in some cases, exhibit a very large electrical conductivity and charge carrier mobility [2]. The research shows that CNTs with semiconductor behavior can be employed as gas sensing materials, exploiting the influence that the adsorption of gases from the environment has on their electronic conductivity. In fact, it was shown that adsorbates can donate or accept free carriers, moreover, they can increase the electronic scattering [8,9,10]. The very high surface to volume ratio and the structural characteristics make CNTs-based materials particularly appealing for gas-sensing applications.

Additionally, CNTs are suitable to be used in combination with different materials such as organic polymers or metallic nanoparticles (NPs), resulting in materials with a significantly improved gas sensitivity and selectivity [11,12]. 

In detail, the gas selectivity and sensitivity performance of CNTs-based sensors has been widely studied and investigated in the literature. The absorption mechanism for several gases such as SO_2_; H_2_S CF_4_ ([13]); and NO_2_, CO, and N_2_ ([8,14]) has been generally studied and modeled by first-principles methods using the density functional theory. Zhao et al. [14] also provided experimental tests validating the theoretical model and showing the influence of the gas induced- charge transfer on the electronic properties of SWCNTs.

Owing to the promising gas-sensing performance predicted by these research works, several kinds of CNTs-based sensors have been realized in different technologies and for multiple applications.

For instance, Sharma et al. realized a resistive ammonia (NH_3_) sensor by screen printing silver electrodes at the terminals of a MWCNTs-based sensing film [15] and flexible substrates such as polytetrafluoroethylene (PTFE) or polyethylene terephthalate (PET) have been used to realize nitrogen dioxide (NO_2_) and hydrogen sulfide (H_2_S) CNTs-based sensors, also working at room temperature [16,17]. Additionally, a volatile organic compound sensor has been realized on paper substrate by the inkjet printing method, exploiting radio-frequency measurements [18]. Appreciable responses have been observed using field emitter transistor (FET) gas sensors based on CNTs for detecting NO_2_ and NH_3_, carbon monoxide (CO), H_2_S, and volatile organic compounds such as ethanol and methanol [19,20,21]. The functionalization of CNTs by metal nanoparticles, metal oxides (MOXs), and different polymers proved to cause an appreciable variation in the electronics properties and to enhance the selectivity and gas sensitivity [13,22,23,24]. Hybrid CNTs–metal oxides-based gas sensors typically show impressive and superior performance with respect to the pristine material, showing a significant reduction of the sensors’ working temperature [25,26,27,28]. Accordingly, nanoparticle-modified gas sensors have been realized by Penza et al., which obtained a very sensitive NO_2_ detection in a mixture of landfill gases using platinum (Pt), ruthenium (Ru), and silver (Ag)-decorated CNTs-based sensors [29]. Furthermore, Au, Ni, and Ti, as well as other metal-nanoparticles, have been used for detecting, for example, benzene (C_6_H_6_) and oxygen (O_2_) [30,31]. 

In this work, we analyze the gas-sensing performance of SWCNTs-based sensors for the detection of NO_2_, as theoretical studies [8] indicate that the adsorption of NO_2_ on many types of SWCNTs is favored with respect to many other gases, and that it is accompanied by a significant charge transfer.

The application contest of the proposed NO_2_ sensors is mainly the industrial scenario, in particular the monitoring of the exhaust emissions of combustion engines in which the NO_2_ concentration can reach levels in the range of tens of ppms [32].

In particular, we propose SWCNT resistive sensors, realized by drop-casting and decorated with nano-particles of both Au and TiO_2_. These materials were selected in the attempt of increasing the selectivity and sensitivity toward the target gas, as well as reducing the operating temperature. In one hand, it was shown [33] that TiO_2_ nanoparticles used as a dopant of p-type nanostructured metal oxide conductive sensors (YCoO_3_) were able to significantly decrease the optimum operating temperature and allowed the development of room temperature operating sensors. On the other hand, Au nanoparticles in conjunction with many different metal oxides were successfully employed to increase the sensitivity toward NO_2_ [34]. 

In this paper, the chemoresistive behavior of the base SWCNT sensor is studied and compared with the ones of sensors exploiting the decorated materials, obtained by adding Au and TiO_2_ nanoparticles to the SWCNTs film. The analysis was conducted by exposing sensor prototypes to gas mixtures of air or nitrogen and NO_2_ or CO, in both dry and humid conditions.

## 2. Materials and Methods

### 2.1. Material Preparation and Characterization

The SWCNTs used in this work were purchased from Sigma-Aldrich (CoMoCat SG65i®, 95% purity, CAS Number 308068-56-6) and used as received. The CNTs have a fraction of semiconducting tubes ≥95%, a fraction ≥40% with (6,5) chirality, and average diameter of 0.78 ± 0.1 nm. 

A dispersion of 0.01 wt.% of the SWCNTs was obtained using a surfactant sodium dodecyl-benzenesulfonate (SDBS) solution. The dispersion was prepared by mixing 7 mg of SWCNTs in 100 mL of aqueous solution of SDBS/DI (deionized) water (1 wt.%). 

The SWCNTs/SDBS aqueous solution was magnetically stirred for several minutes and finally sonicated for 45 min with a spherical tip at 25 °C to ensure the dispersion of the SWCNTs in the liquid phase. A sonication power of 60 W was applied with square pulses (50% duty cycle) using Bandelin Solopuls HD 3200.

Electrical and optical properties were investigated using ad hoc films prepared depositing the SWCNTs colloid on appropriate substrates.

Ultra-violet (UV), visible (VIS) and near infra-red (NIR) measurements were performed using a Perkin-Elmer (Lambda 900) spectrophotometer. 

Scanning electron microscopy (SEM) imaging was carried out by FEI Novanano SEM 200 using SWCNTs films deposited on silicon wafers, sputtered by platinum to increase the conductivity, and hence improve the image quality.

Atomic force microscopy (AFM) imaging was performed by Keysight 5600 L. To be able to image the CNTs’ distribution, thin films of SWCNTs were used. These were obtained by depositing the SWCNTs on silicon substrates and by spin coating at 6000 rpm to have a very thin film.

The SWCNT-based material was decorated by means of depositions of liquid colloids of Au and TiO_2_ nanoparticles.

Au nanoparticles with 5 nm of diameter are dispersed in a stabilized suspended solution of phosphate buffered saline (PBS) with a concentration equal to 5.5 × 10^13^ particles/mL. The colloid was purchased from Sigma Aldrich and used as it stands.

The TiO_2_ nanoparticle colloid in water was purchased from Italvernici (ITALVERNICI-FELCE150) and it is based on crystalline anatase nanoparticles with diameters in the range of 25–55 nm diluted in water with a concentration of 32 × 10^−3^ mol/L.

### 2.2. Sensor Realization and Gas Sensing Characterization System 

**Figure 1:**
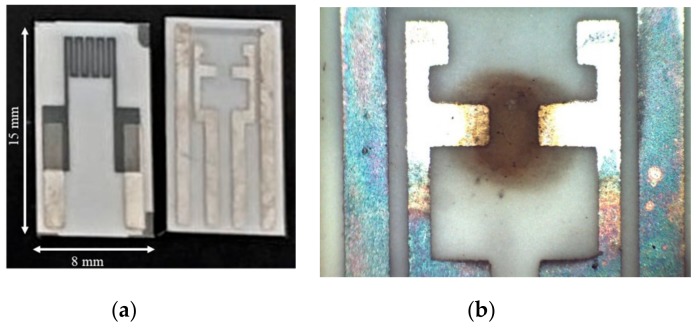
Back and front view of the used sensor structure (**a**) and microscopic image of the deposited chemical sensing film (**b**).

The gas sensors prototypes were realized on alumina substrates, as shown in Figure 1, depositing thin sensing films across two silver electrodes realized by screen printing technology, by drop-casting 1 μL doses of the dispersion of SWCNTs described in the preceding section with a micropipette. The sensor films cover an area between the two electrodes of approximately 7.5 mm^2^. 

The obtained samples were heated at 400 °C for 24 h; in Figure 1b, a picture of the resulting device is reported.

Sensors based on decorated materials were obtained by drop casting the solutions of Au or TiO_2_ in doses of 1 μL over the SWCNT film, drying in ambient air, and then heating at 400 °C for 4 h. Sixteen SWCNT sensors were prepared and tested. Four of them were decorated by subsequent TiO_2_ depositions, whereas four were decorated by subsequent Au nanoparticle depositions. The sensors are additionally provided with a Pt-based temperature sensor and a heater on the back side (Figure 1), which allow to perform an accurate control and real-time monitoring of the operating temperature during measurements.

In order to study the gas sensing performance of the realized devices, the variation of the sensing film conductance is acquired in different environmental conditions in terms of gas concentration and species, operating temperature, and humidity.

The automatic measurement system used in this work provides the possibility of simultaneously characterizing up to eight sensors that are arranged in an ad hoc designed steel measurement chamber [35]. The system allows for digitally controlling and managing the measurement temperatures up to 400 °C with an accuracy lower than 3 °C. The gas flow is constant during the measurements and equal to 200 mL/min for this work; different gas concentrations are obtained using a digitally controlled gas flow meter system (BronkHorst F-201C). Humid flows are obtained by means of a bubbler containing ultrapure water, which is kept at a known and constant temperature, used to obtain a saturated flow of the carrier gas. The humid flow is then mixed with a dry flow so to obtain the desired relative humidity (RH) value. The tests described hereafter were conducted at RH equal to 25% (at 25 °C).

During the measurements, the temperature, the humidity in the measurement chamber, and the gas concentration are varied according to different measurement profiles. The sensors were tested in mixtures of air, nitrogen, and NO_2_ or CO in dry or humid flows. Each test was repeated three times, to assess the repeatability.

## 3. Results and Discussion

### 3.1. Physical Characterization

The analysis reported in this section aims to characterize the dispersion of the SWCNTs prepared by the authors and the uniformity of the films by deposition.

**Figure 2:**
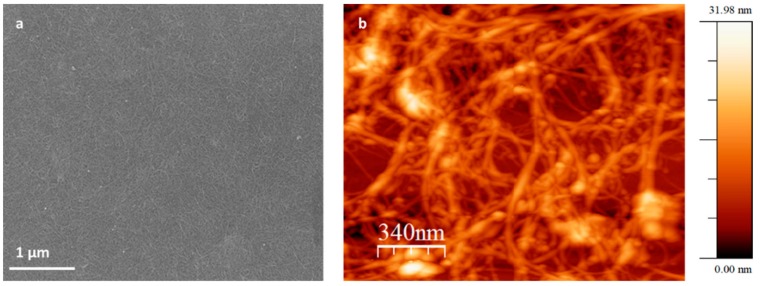
(**a**) Scanning electron microscopy (SEM) image of the single wall carbon nanotubes (SWCNTs) casted on Si wafer and (**b**) atomic force microscopy (AFM) image of the SWCNT spin-coated on Si substrate.

Figure 2a reports the SEM image confirming the uniform dispersion of the SWCNTs films. The SWCNTs film is also shown in Figure 2b, where the AFM image also shows a well-distributed network, which confirms the good dispersibility of the SWCNT. The image shows a roughness of a maximum of 32 nm, which is partially justified by the sample preparation procedure (high-speed spin-coating). In Figure 2b, the individual SWCNTs (whose diameter is ~1 nm) appear to be quite inflated. This could be explained by the possible formations of bundles or by the coating with sputtered Pt and the contrast between conductive tubes and insulating neighboring areas [36]. 

**Figure 3:**
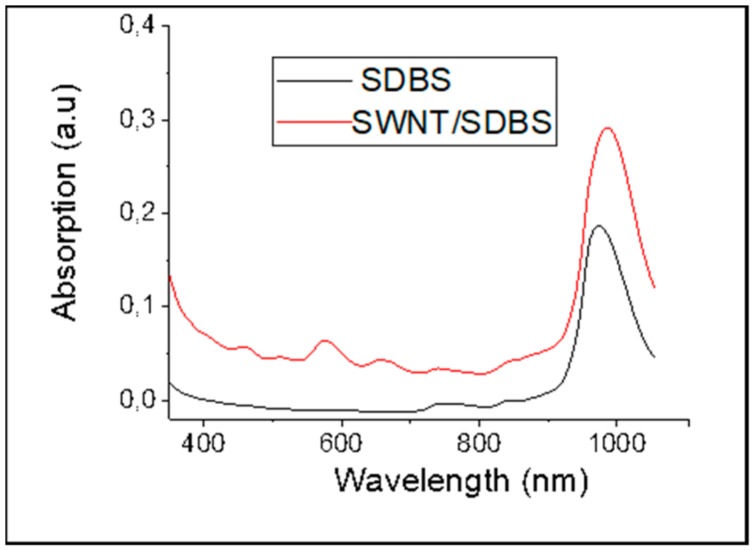
UV/VIS/IR absorption spectrum for SWCNTs dispersions. SDBS, sodium dodecyl-benzenesulfonate.

The UV/VIS/NIR spectrum for SWCNTs dispersion was measured by diluting them by 30 times in the aqueous SDBS solutions. It shows a peak response at wavelengths S_11_ at 565 nm in the visible range and S_22_ from 970 nm to 1010 nm (peak at ~985 nm), which can be attributed to semiconducting nanotubes in the NIR region [37], as shown in Figure 3. The measurements also revealed a wide spectral response from visible to the near-infrared corresponding to different chirality of the CNTs. Both imaging and UV/VIS/NIR spectra prove the successful dispersion and homogeneity of the film, at least at the micro and macro scale. 

### 3.2. Gas Measurement Results

In this section, the proposed sensors are characterized experimentally in terms of baseline resistance, of response to the target gas, and finally of response time. In this section, the sensor performances are analyzed using the following definition of response:

(1) $$Resp=\frac{G-G_{0}}{G_{0}},$$

where *G_0_* is the steady state value of the conductance in the carrier gas at the working temperature, and *G* is the conductance value at the same temperature.

The experimental results presented hereafter refer to the response evaluated after 4 min of exposure to the test mixture. In the case that the steady state is not reached in the selected exposure time, the response according to Equation (1) underestimates the steady state response. This definition was used because it also accounts for the sensor response time. The exposure time was selected to penalize those behaviors that are very slow and are thus related to combinations of temperature/material/target gas, which are hardly usable in practical situations.

The dynamical behavior of the sensor is hard to be described by few parameters, as it is well known that the adsorption/desorption phenomena are generally non-linear. In this paper, the sensor reactivity is analyzed simply considering the response time (which is the time needed for the response to go to 90% of the steady state value when the gas flow in the measurement chamber is suddenly switched from pure carrier to target gas mixture) and the recovery time (which conversely measures the time needed to change from 90% of the response to the steady state value when changing the gas flow composition from target gas mixture to the pure carrier gas). Obviously, both times depend on the temperature and mixture composition (e.g., NO_2_ concentration). 

The experimental results shown hereafter concern the typical behaviors of the different sensors. The repeatability obtained with the same sensors is about 20% of the response value (Equation (1)) and the reproducibility is about 30% of the response value.

### 3.3. Study of the Baseline Conductance

The baseline conductance of the different sensors was studied in order to gain insight into the electronic conductivity, to better understand the gas sensing mechanism, and to get some information about the effects obtained by the deposition of the NPs on the SWCNTs films. Moreover, this subsection aims at characterizing the dependence of the sensor conductance on some relevant environmental conditions. 

In particular, the baseline conductance was measured in steady state condition in dry and humid nitrogen (inert gas) and air, in the temperature range of 120–240 °C. In the tested temperature range, the sensing films have conductance values in the range of 1 × 10^−5^ S–1 × 10^−4^ S, which is a convenient interval for low-cost measurements.

Some preliminary tests were conducted by performing subsequent depositions on pristine sensor substrates of the same doses used for surface decorations with the suspension of Au nanoparticles. It was found that no measurable resistance could be read by the characterization system in the tested temperature range; consequently, the conductance of the as-obtained films, *G_f2_*_,_ is smaller than 10^−10^ S. On the other hand, the deposition of TiO_2_ causes the formation of a film with a conductance *G_f2_* lower than 10^−7^ S. So, in both cases, the measured conductance values were negligible with respect to those of the SWCNT films.

**Figure 4:**
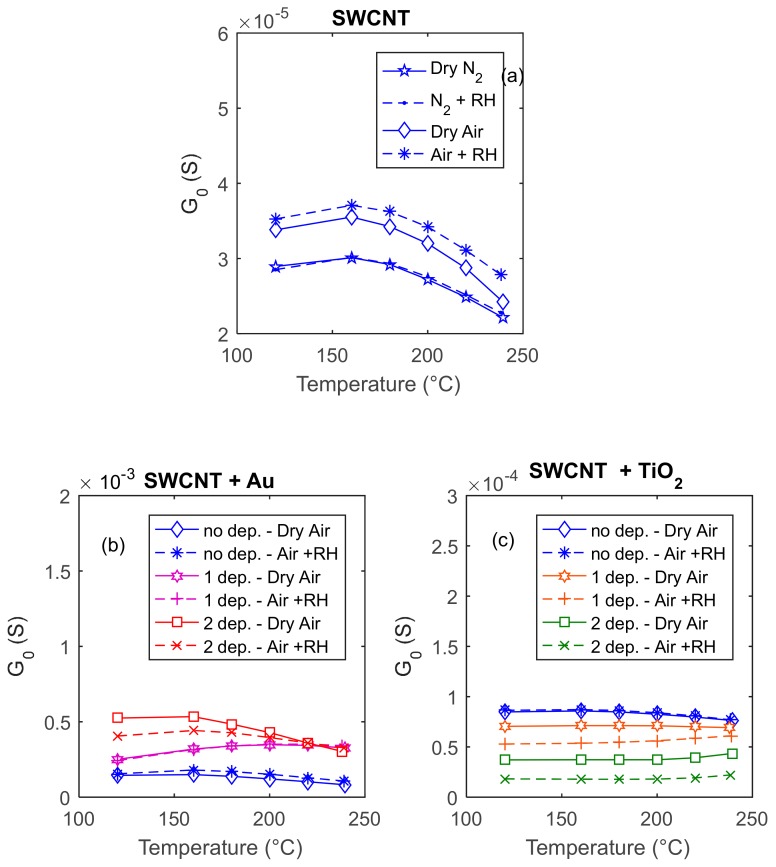
Dependence of the baseline conductance on temperature, in dry nitrogen (N_2_), dry synthetic air, humid nitrogen (relative humidity (RH) 25% @25°C), and humid air (RH 25% @25°C). In plots (**a**), (**b**), and (**c**), the blue lines represent the baseline conductance of three sensors obtained with a deposition od SWCNTs, whereas the colored lines in the lower plots (**b**) and (**c**) represent the response of the same sensors after subsequent depositions of Au (left) and TiO_2_ (right), as per the legend.

In Figure 4, the behavior of the baseline conductance is shown as a function of the working temperature in different conditions for three different sensors, two of which were characterized, decorated with Au (Figure 4b) or TiO_2_ (Figure 4c) nanoparticles depositions, and finally characterized with the same measurement protocols after the decoration. It can be seen that the deposition of NPs causes a large effect on the baseline resistance of the sensors in all the tested conditions. This can be considered as an indication that the added material actually modifies the electronic/chemical behavior of the existing film. In fact, if the deposition of nanoparticles resulted in the formation of a new conducting film on top of that of the previous SWCNTs, then the sensing film would have been simply the parallel of the two films and the baseline conductance of the obtained composite film, *G_0_*, should always be larger than that of the SWCNTs, being the following:

(2) $$G_{0}=G_{f10}+G_{f20},$$

where *G_f10_* is the conductance of the existing SWCNTs film and *G_f20_* is the baseline conductance of the added film. Instead, it can be seen that, in the case of the TiO_2_ depositions, the conductance of the baseline decreases, whereas in the case of Au nanoparticles, it increases, but of an amount that is not explainable with the simple contribution of the parallel conductance $G_{f20}$. Hence, the effect of deposing a dose of NPs is not simply that of contributing with a new film in parallel with the first one.

The pure material is expected to behave as a p-type semiconductor, owing to intrinsic acceptor-type defects; therefore, an increased conductivity is expected in air, with respect to the one measured in nitrogen, because oxygen adsorption is accompanied by negative charge transfer (extrinsic acceptor state creation) [9]. In accordance with the expected behavior, it can be seen from the results (Figure 4a) that the presence of oxygen causes an increase of the baseline conductance. It must be noted that the effect is minor, showing a limited influence of oxygen adsorption on the electronic conduction, despite the significant adsorption energy for oxygen and large charge transfer predicted by some studies [38].

Moreover, there is a little difference in the results obtained in dry and humid environments, showing a very slight sensitivity to humidity. This result is in accordance with the theoretical studies [9], which predict a small sensitivity for water; this latter is also accompanied by a small charge transfer (which can be positive or negative depending on the tube chirality). In the tested material, water increases the conductivity, which indicates a negative charge transfer (acceptor behavior), in contrast with what happens with the most part of metal oxides. Moreover, it was experimentally confirmed that, at low humidity (RH below 40%), the change in resistivity is very low [39].

The observed behavior of the baseline conductance as a function of temperature (which is the same in presence or in absence of adsorbed oxygen) is different from the typical behavior found for MOX (especially p-type), which essentially describes a thermal activated conduction. In fact, at temperatures larger than about 120 °C, the conductivity decreases with temperature, probably because of the reduction of mobility. This observation can be related to other results in the literature, which explain the influence of adsorbed gases on the electronic behavior of CNTs not only by charge transfer (chemisorption), but also by the change in the electron and hole-free carrier lifetimes (or, equivalently, the carrier mobility). Large changes in the carrier lifetime were observed in the presence of physiosorbed gases (which, in this case, could be nitrogen or oxygen), which can be caused either by the increased carrier scattering from dynamic defect states, associated with momentarily adsorbed gases, or by non-thermal localized SWCNTs phonons generated by collisions of the gas molecules with the tube wall.

A different behavior is observed for the NP-decorated sensors. The addition of TiO_2_, which is an n-type material, could in principle deeply modify the electronic behavior of the sensing film. The composite obtained could be characterized by the appearance of p-n hetero-junctions, and the gas affinity can be highly modified by the presence of a chemically reactive material as TiO_2_. TiO_2_ addition causes a decrease of the baseline conductivity and significantly enhances the sensitivity to humidity, as expected, as water adsorption on TiO_2_ is highly favored (Figure 4c) [33]. On the contrary, the addition of Au nano-particles, which also makes free electrons available, has the unexpected effect of increasing the conductance of the film. Meanwhile, the sensitivity to water vapor remains very small in case of a single deposition and becomes large at low temperature when using a larger quantity of Au (2 depositions, Figure 4b). In all tested cases, the NP-decorated sensors show measurable and stable baseline values.

**Figure 5:**
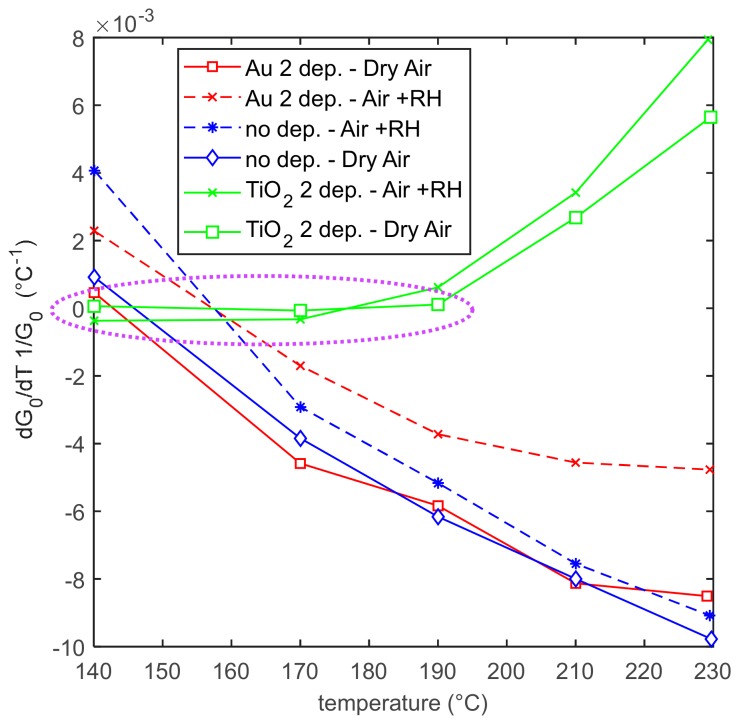
Relative sensitivity of the sensor conductance toward temperature (temperature coefficient of conductance) as a function of temperature for the different sensors types and in different gaseous environments.

The assessed relative sensitivity of the baseline conductance toward temperature, usually called the temperature coefficient of conductance, is shown in Figure 5. It can be seen that it has acceptable values (always lower than 10^−2^ °C^−1^), and for TiO_2_ decorated sensors, there is a large temperature interval (140–190 °C), where this coefficient is very low. This scarce sensitivity toward temperature makes the application of these sensors easier. In fact, in the case of a large sensitivity on temperature (as for many metal oxides, which show a purely thermally activated conduction, that is, exponential dependence on temperature), the working temperature has to be held very stable requiring complex control systems and electronic front-ends; otherwise, the response to gases can be masked by the effect of working temperature fluctuations.

### 3.4. Study of the Response to NO_2_

**Figure 6:**
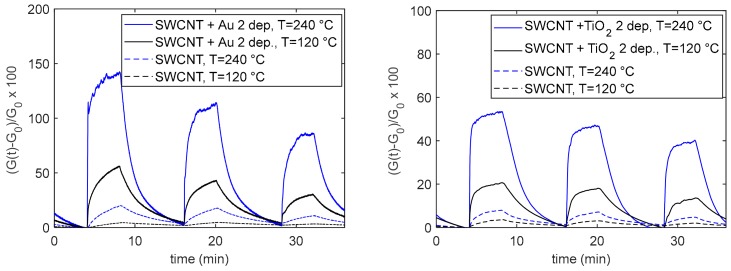
Typical response of the base SWCNTs and nanoparticle (NP)-decorated sensors. The measurement protocol is as follows: 4 min carrier gas, 4 min carrier gas + 50 ppm NO_2_, 8 min carrier gas, 4 min carrier gas + 25 ppm NO_2_, 8 min carrier gas, 4 min carrier gas + 12.5 ppm NO_2_, 4 min carrier gas. Dry mixtures, carrier gas air.

In this subsection, the response to NO_2_ of the base SWCNT sensor is assessed; moreover, it is shown how the addition of NPs positively modifies the NO_2_ sensing, as can be noticed from the experimental results shown in Figure 6. The plots show the typical responses (according to Equation (1)) of the different sensor types to NO_2_ mixture pulses, at two different and constant working temperatures, as a function of time. 

The concentrations of the NO_2_ mixtures used for the tests presented in this section were selected with the aim of evaluating the applicability of these materials, for instance, in the context of combustion monitoring in engines [40,41].

**Figure 7:**
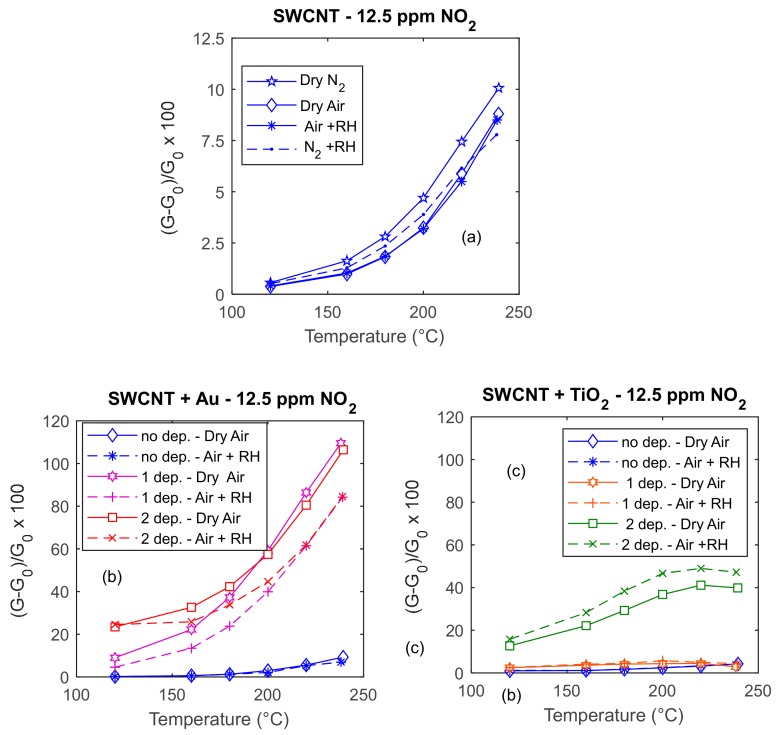
Responses (according to Equation (1)) to NO_2_ of three different sensors as a function of temperature; the NO_2_ concentration is 12.5 ppm, dry and humid air and nitrogen (RH = 25% @25 °C) are used as carrier gases. In plots (**a**), (**b**), and (**c**), the blue lines represents the response to 12.5 ppm of NO_2_ of three sensors obtained with a deposition of SWCNTs, whereas the colored lines in the lower plots (**b**) and (**c**) represent the response of the same sensors after subsequent depositions of Au (left) and TiO_2_ (right), as per the legend.

In Figure 7, the responses to 12.5 ppm of NO_2_ of three different sensors are presented as a function of temperature in the range of 120–240 °C. The results concern tests with different carrier gases (dry and humid air). In the plots, the blue lines represent the response to 12.5 ppm of NO_2_ of the three sensors obtained with a deposition of SWCNTs, whereas the colored lines in the lower plots represent the response of the same sensors after subsequent depositions of Au or TiO_2_. The results allow for evaluating the effect of surface decoration on an individual specimen, avoiding issues related to reproducibility, which is limited to about 20% with this prototyping route.

As expected, on the basis of previous experimental works and from theoretical studies [9], the response to NO_2_ is the one observed in other p-type semiconductors in all the tested conditions. In detail, the adsorbed molecule behaves as an acceptor that, by trapping free electrons [9], creates additional holes available for the conduction, so that the exposure to NO_2_ causes an increase of the tube conductivity. This observation is supported by the theoretical work of Peng and Cho [42], who studied the adsorption of NO_2_ onto SWCNTs by first-principles calculations using density functional theory (DFT); that is, NO_2_ is found to bind with SWCNTs with an adsorption energy of 0.3 eV, and it is also found that the molecule has high diffusion kinetics on nanotubes’ surfaces. The electron density analysis shows that charge transfer is induced from C atom to the NO_2_ gas molecule leading to hole (or p-type) enhancement of semiconducting (10, 0) nanotubes. On analogy of what happens with MOX nano-structured films, NO_2_ adsorption traps electrons on the surface; therefore, as already highlighted, it causes an increase of free carriers available, but it also creates an electric field on the surface, which interferes with the motion of free carriers from a tube to the neighbor one. Also, this effect is very important in determining the overall conductance of the film, which is composed by a large number of CNTs. 

The experimental results in Figure 7 show that the sensitivity of the base material increases with temperature in the whole tested range where the optimum temperature is not reached (>240 °C). The maximum sensitivity to NO_2_ is lower than 1%/1 ppm at 240 °C. Moreover, it can be seen that the response to NO_2_ of the SWCNT film is almost insensitive to the presence of humidity when operating in air.

Looking at Figure 7c, it can be seen that the addition of TiO_2_ does not change the electronic behavior of the overall film, which still behaves as a p-type semiconductor. Moreover, it significantly increases the sensitivity, which reaches about 4%/ppm at 240 °C. As expected, the effect of TiO_2_ is particularly important at low temperature; for example, at 180 °C, the ratio between the response of the same sensor with and without added TiO_2_ is about 20. This result is in accordance with other research outcomes [37], showing how the addition of TiO_2_ activates low temperature responses. For the decorated material, the optimum temperature is 220 °C. As a drawback, the presence of TiO_2_ increases the cross-sensitivity to RH.

Finally, looking at the results shown in Figure 7b, it can be seen that the addition of Au NPs dramatically increases sensitivity; in particular, at 240 °C, the sensitivity is increased by a factor of 10, but the gain is far larger at lower temperature. It is worth noting that at high temperature successive depositions do not further improve the sensor sensitivity, whereas at lower temperatures, an increase in the Au quantities is accompanied by an enhancement of the sensor response. As a drawback, also in the case of Au addition, the cross-sensitivity to water is increased with respect to the base material.

The presented results, which show a strong influence of NPs’ addition, further enforce the assumption that the deposition of NPs results in an actual modification of the base SWCNTs’ material and tends to exclude the formation of an NP film on top of the pre-existing SWCNT film. In fact, the response of a sensor consisting of the combination of two films in parallel is determined by the film with the dominant conductance, hence the addition of a material with a negligible conductance would lead to negligible modifications of the sensor response, as can be inferred from the following equation:

(3) $$\mathrm{Resp}=\mathrm{Res}p_{1}\frac{G_{f10}}{G_{0}}+\mathrm{Res}p_{2}\frac{G_{f20}}{G_{0}},$$

where $Resp_{1}=\frac{\left(G_{f1}-G_{f10}\right)}{G_{f10}}\;\;$ is the response of the lower film, and $Resp_{2}=\frac{\left(G_{f2}-G_{f20}\right)}{G_{f20}}\;$ is the response of the film on top. Hence, obviously, if we have, as in our cases, $G_{f10}\gg G_{f20},\;\;$ we would have $Resp\approx Resp_{1}\;$. So, especially, the addition of Au nanoparticles would give a negligible contribution to the sensor response.

**Figure 8:**
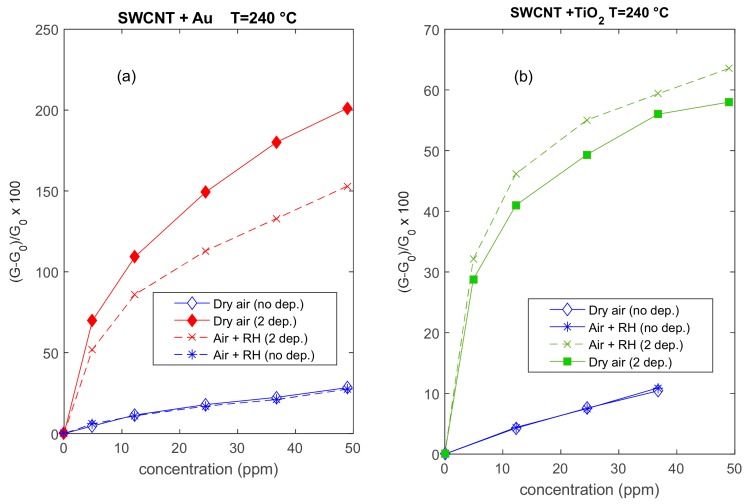
Responses (according to Equation (1)) to NO_2_ of two different sensors as a function of the NO_2_ concentration; the working temperature is 240 °C, dry and humid air (RH = 25% @25 °C) are used as carrier gases. The blue lines represent the response of the two sensors with a deposition of SWCNTs, whereas the colored lines represent the responses of the same sensors after subsequent depositions of Au (**a**) and TiO_2_ (**b**), as per the legend.

In Figure 8, the responses of the different type sensors are plotted as a function of NO_2_ concentration in different carrier gases. Au-decorated sensors apparently show a larger ‘linear range’.

**Figure 9:**
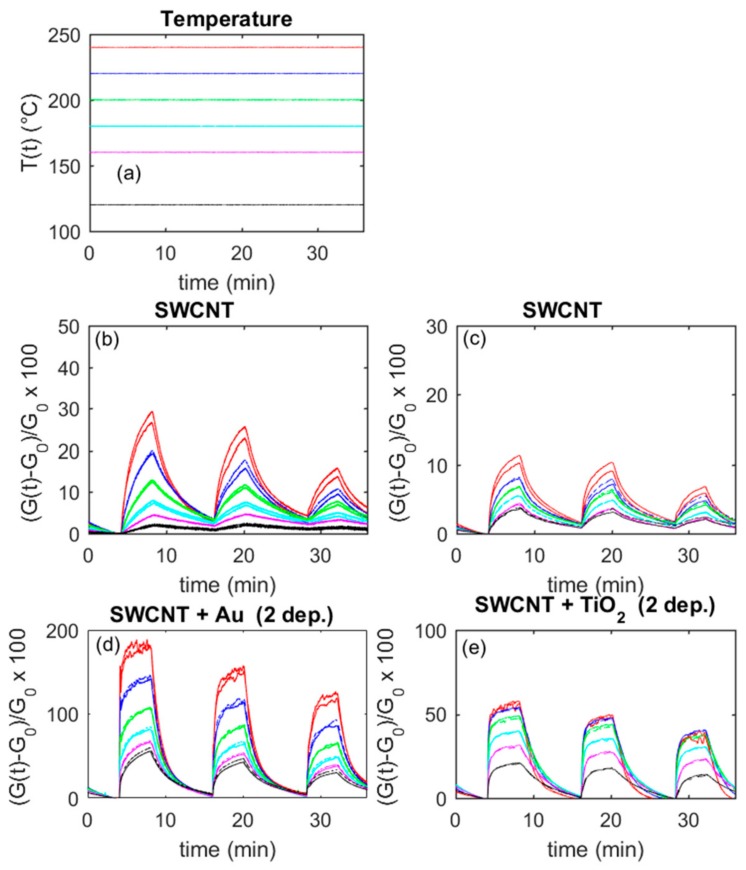
Responses (according to Equation (1)) to NO_2_ of two different sensors as a function of time; different colors represent responses obtained at the different working temperatures shown in the upper left plot (**a**). Dry air is the carrier gas. The upper plots (**b**) and (**c**) represent the responses of two sensors obtained with a deposition of SWCNTs, whereas the lower plots (**d**) and (**e**) represent the responses of the same sensors after two depositions of Au (left) and TiO_2_ (right). (**d**) Plots refer to the NPs addition on the sensor whose responses are represented in (**b**), whereas (**e**) plot corresponds to (**c**). The measurement protocol is as follows: 4 min carrier gas, 4 min carrier gas + 50 ppm NO_2_, 8 min carrier gas, 4 min carrier gas + 25 ppm NO_2_, 8 min carrier gas, 4 min carrier gas + 12.5 ppm NO_2_, 4 min carrier gas.

In Figure 9, some examples of the transient response to NO_2_ concentration abrupt changes are shown as a function of time. The plots in the figure allow for a comparison of the response of two sensor specimens in the same conditions, before (upper plots) and after the addition of NPs (lower plots). It can be seen that the addition of both TiO_2_ and Au nanoparticles significantly reduces the sensor response time. In fact, the base material is characterized by response times larger than 4 min and recovery times larger than 8 min, even at 240 °C, whereas the Au addition (two depositions) reduces the response time to about 0.6 min and the recovery time to about 4 min (at 240 °C, 50 ppm NO_2_). Furthermore, TiO_2_ addition (two depositions) leads to a response time of 1 min and a recovery time of 3 min (at 240 °C, 50 ppm NO_2_).

**Figure 10:**
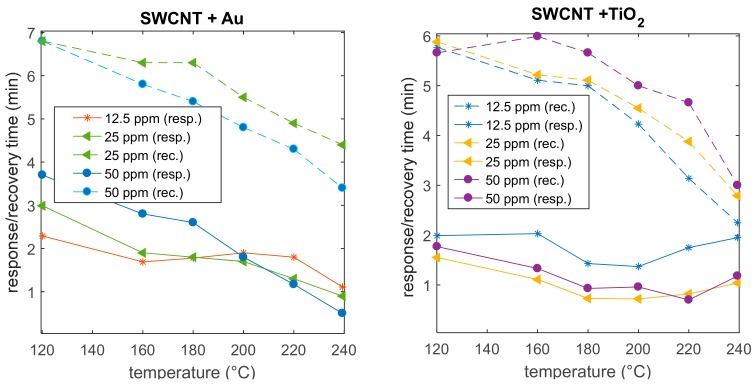
Response (solid lines) times and recovery (dashed lines) times (defined in Section 3.2) relative to the transient responses shown in Figure 9 as a function of temperature for two sensors decorated with Au (left) and TiO_2_ (right).

The estimated response and recovery times, corresponding to the transient responses shown in Figure 9, are plotted in Figure 10. For Au-decorated SWCNTs, as usual, the response and recovery times significantly increase with the decreasing temperature, so that low working temperatures are not practical for real-world applications. On the opposite hand, in the case of TiO_2_ addition, there is a larger temperature interval within which the sensor response time is satisfactory.

### 3.5. Low Temperature Operations

In this subsection, some experimental results are presented, aiming at assessing the NO_2_ sensing properties of the proposed sensors at a low temperature, to explore the possibility of using them in simple low-cost, low-power systems without any heating, in the perspective of room temperature (RT) operations.

At RT, the adsorption is favored, while the charge exchange (chemisorption) is less probable or can be very slow. Usually, its kinetics can be dramatically modified by the presence of dopants that can act as catalysts; therefore, in this sub-section, the experimental assessment of the decoration effect is investigated. To obtain these data, the sensors were operated without heating the substrates and placed in the test chamber in an environment where the temperature was not controlled. During the measurements, the temperature spanned an interval of about 15 °C from approximately 35 °C to 50 °C. The same measurement protocols described in the previous subsections were used. 

**Figure 11:**
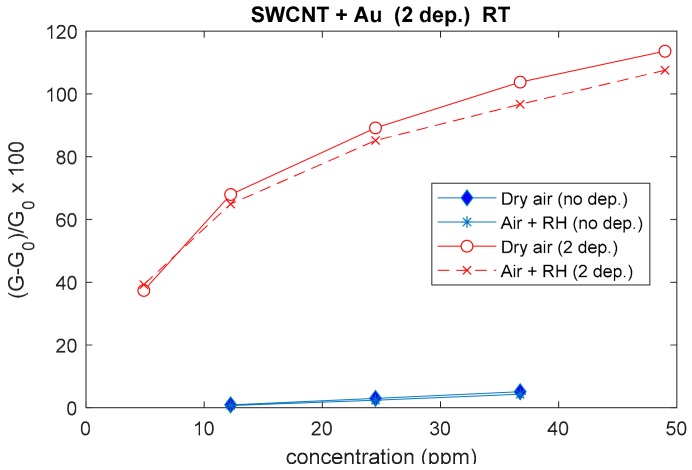
Responses (according to Equation (1)) toward NO_2_ of an Au-modified sensor as a function of NO_2_ concentration, obtained with no heating (40 °C) in dry and humid air carrier gases. Blue lines represent the response of the sensor with a deposition of SWCNTs, whereas the red lines represent the response of the same sensor decorated with two depositions of Au nanoparticles.

**Figure 12:**
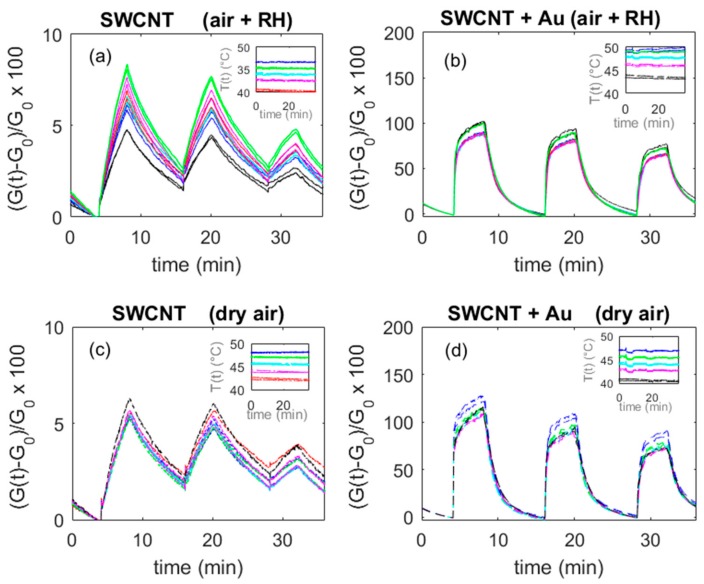
Responses (according to Equation (1)) to NO_2_ of an Au-decorated sensor as a function of time. Different colors represent responses obtained at the temperatures plotted in the insets. Results of 10 repeated tests with not controlled temperature, which varied in a 15 °C range during tests (as can be seen in the insets). Dry air and humid air (25% at 25 °C) are the carrier gases. Plots (**a**) and (**c**) represent the responses of the same sensor obtained with a deposition of SWCNTs ((a) humid air; (**c**) dry air), whereas the right plots represent the responses of the same sensor after two depositions of Au in the same conditions ((**b**) humid air, (**d**) dry air). The measurement protocol is as follows: 4 min carrier gas, 4 min carrier gas + 50 ppm NO_2_, 8 min carrier gas, 4 min carrier gas + 25 ppm NO_2_, 8 min carrier gas, 4 min carrier gas + 12.5 ppm NO_2_, 4 min carrier gas.

In Figure 11 and Figure 12, some results are presented. The base material shows a measurable response with magnitudes close to those found at higher temperatures; nevertheless, the response and recovery times are very long, making the application of this material for RT operations impractical. On the other hand, it was found that the deposition of both TiO_2_ and Au nanoparticles enhances the sensor performance significantly, as it can be seen in Figure 11, where data concerning the Au NPs addition are presented. In this figure, the response is evaluated as the average value of three measurements performed at 40 °C. The comparison with the base material transient responses (in Figure 12) shows that the response is greatly enhanced, owing to an increased sensitivity, but also to the outstanding increase of the reaction rate.

TiO_2_ decorated sensors show a large variability of the response. This was explained by the large sensitivity to humidity shown by this material at room temperature [43], which seems to also heavily affect the reactivity of the sensor toward NO_2_. On the other hand, Au-decorated sensors show a stable behavior, almost insensitive to humidity at low temperatures, in contrast with what was observed at higher temperatures (compared with data shown in Figure 7). Finally, the response sensitivity to temperature is limited, about 2%/°C in the tested interval range.

### 3.6. Cross-Sensitivity toward CO

Some tests were performed using measurement protocols similar to those described in the previous subsections to characterize the response toward NO_2_ with concentration of CO up to 2000 ppm. In all the tested conditions, no response distinguishable from noise was recorded.

## 4. Conclusions

The SWCNTs sensors presented in this work show a limited response to NO_2_, and are insensitive to RH, but are too slow to be easily applicable. The addition of nanoparticle of TiO_2_ and of Au, via a facile route of preparation, is able to tremendously increase the performance of the sensor, allowing for reaching large sensitivities. One deposition of Au reaches 10%/ppm (at 12 ppm NO_2_, 240 °C), while two depositions of TiO_2_ allow to reach 3.7%/ppm (at 12 ppm NO_2_, 200°C). Both decorations significantly decrease the response and recovery times of the sensors: Au nanoparticles’ addition allows for reaching response times of 0.5 min at 240 °C, whereas TiO_2_ addition improves performance, especially at lower temperatures; for example, at 180 °C, the response time is still about 1 min (25 ppm NO_2_). Moreover, at this temperature, 180 °C, the temperature coefficient of conductance of TiO_2_ added films is very small (< 10^−4^ °C^−1^), allowing for stable operations in low-cost systems. On the other hand, the proposed Au addition leads to better performance at low temperatures in terms of immunity to RH and of sensitivity (5%/ppm @12 ppm NO_2_, 40°C), and a satisfactory stability. These sensors also open the possibility of operations at RT.

In conclusion, we have demonstrated a facile preparation route for the realization of SWCNT-based sensors and the enormous improvements that can be obtained with modifications performed using commercial materials and low-cost and simple processes.
